# Oxford unicompartmental knee arthroplasty: medial pain and functional outcome in the medium term

**DOI:** 10.1186/1749-799X-6-52

**Published:** 2011-10-10

**Authors:** Mark C Edmondson, David Isaac, Malin Wijeratna, Sean Brink, Paul Gibb, Paul Skinner

**Affiliations:** 1 Kent and Sussex Hospital, Mount Ephraim Rd, Tunbridge Wells, Kent, TN4 8AT, UK

## Abstract

**Background:**

In our experience results of the Oxford unicompartmental knee replacement have not been as good as had been expected. A common post operative complaint is of persistent medial knee discomfort, it is not clear why this phenomenon occurs and we have attempted to address this in our study.

**Methods:**

48 patients were retrospectively identified at a mean of 4.5 years (range = 3 to 6 years) following consecutive Oxford medial Unicompartmental Knee arthroplasties for varus anteromedial osteoarthritis. The mean age at implantation was 67 years (range 57-86). Of these 48 patients, 4 had died, 4 had undergone revision of their unicompartmental knee replacements and 2 had been lost to follow up leaving 38 patients with 40 replaced knees available for analysis using the 'new Oxford Knee Score' questionnaire. During assessment patients were asked specifically whether or not they still experienced medial knee discomfort or pain.

**Results:**

The mean 'Oxford score' was only 32.7 (range = 16 to 48) and 22 of the 40 knees were uncomfortable or painful medially.

The accuracy of component positioning was recorded, using standard post operative xrays, by summing the angulation or displacement of each component in two planes from the ideal position (according to the 'Oxford knee system radiographic criteria'). No correlation was demonstrated between the radiographic scores and the 'Oxford scores', or with the presence or absence of medial knee discomfort or pain.

**Conclusion:**

In our hands the functional outcome following Oxford Unicompartmental knee replacement was variable, with a high incidence of medial knee discomfort which did not correlate with the postoperative radiographic scores, pre-op arthritis and positioning of the prosthesis.

## Background

There have been impressive survivorship studies, from both originator and non originator data, for the Oxford Unicompartmental Knee prosthesis, with rates of 94-100% at 10 years, and 95% at 14 years [1-5] and 90% at 15 years [6]. There are fewer studies describing the functional outcomes of this prosthesis [7-9]. Van Isaker et al found that 79% rated as 'excellent' or 'good', with 10.5% moderate and 10.5% poor results following replacement with an Oxford prosthesis in 65knees (using the HSS score, average score 164). Cottenie et al demonstrated 80% excellent, 10% good, 4% fair, 6% poor results in 69 knees (mean HSS score 178).

In our experience the results of the Oxford medial unicompartmental knee arthroplasty have been variable. Although the incidence of persistent medial knee pain post Oxford unicompartmental replacement has been quoted as approximately 1% [10], we found this to be a common complaint in our patients with poorer results. We hypothesised that this may be due to malpositioning of the tibial tray and particularly excessive medial overhang.

We studied patient satisfaction in the medium term. We also investigated whether functional scores and medial pain correlated with the positioning and alignment of the prosthesis when assessed radiographically (using the postoperative radiographic criteria listed in the Oxford unicompartmental knee replacement surgical technique manual) [11].

## Methods

Our study took place in a busy district hospital Orthopaedic department which performs on average 180 TKRs a year, with good published outcomes [12]. Between August 2000 and August 2004 48 Oxford Unicompartmental Knee Arthroplasties were performed, and these were identified at a mean of 4.5y (range 3-6y) following surgery. (These were the 'Phase III' - using old style numeric tibial trays and standard bracket non anatomic meniscal bearings through an MIS approach). Very strict inclusion criteria were adhered to in the selection of the patients for UKA, as set out by Goodfellow et al [13], and in addition patients with significant patellofemoral osteoarthritis were excluded.

All patients that underwent Unicompartmental knee replacement had significant anteromedial Osteoarthritis, of these 30 of the 48 had radiographic Grade 4 (bone on bone) arthritis, the remaining 18 had grade 3 OA.

Of the 48 patients, four had undergone revision, four had died since implantation and 2 could not be traced.

**Figure 1:**
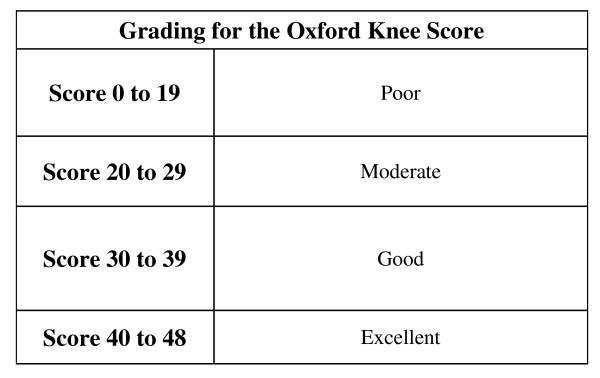
**Oxford knee score**.

The remaining 38 patients responded to a postal and telephone enquiry using the Oxford Knee Score functional questionnaire [14] - where 0 is the worst score and 48 the best. Scores of 0-19 as 'poor', 20-29 as 'moderate', 30-40 as 'good' and 40-48 are perceived as 'excellent' (Figure 1). Patients were specifically asked about the presence or absence of medial knee discomfort or pain. This was done in the postal enquiry by showing a diagram of a knee and asking patients to report where (if at all) they experienced *persistent *pain or discomfort by placing a cross on the diagram at the area of maximal discomfort. During the telephone assessment patients were asked - "which part of your knee is painful (if at all)?" Patients then described the area of discomfort, which was recorded.

**Figure 2:**
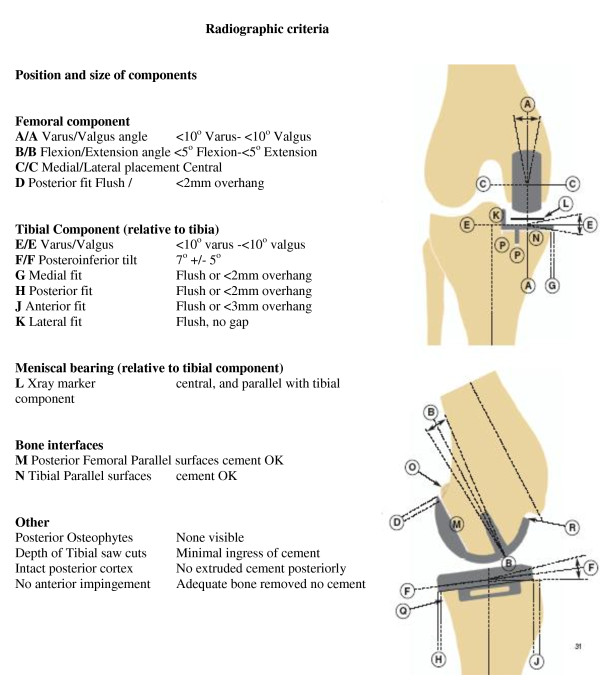
**Radiographic criteria for optimal positioning of the Oxford Unicompartmental Knee replacement**.

All patients had their postoperative radiographs compared to the radiographic criteria listed in the 'Biomet surgical technique' manual (Figure 2). Each angle was recorded together with the degree of overhang of the prosthesis in millimeters and the presence or absence of posterior osteophyte.

Each prosthesis was then scored radiographically by summing the degree of deviation of the implant from perfect alignment in two planes, and adding the overhang in mm and the presence of posterior osteophyte (present = 1, absent = 0). For example a tibial tray with varus alignment of 6 degrees, a 2 mm medial overhang and posterior osteophyte would achieve a score of 9.

**Figure 3:**
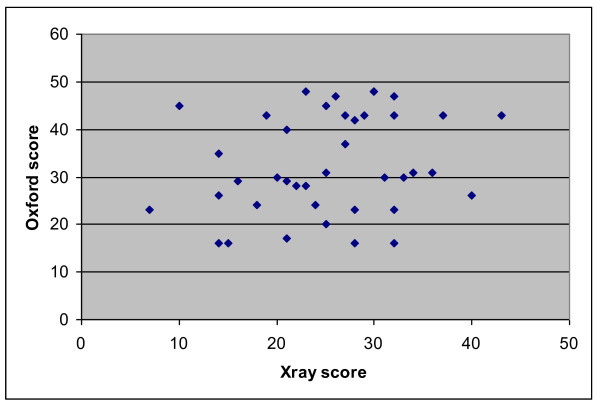
**Scatterplot showing Oxford scores against Postoperative Xray scores**.

The radiographic scores are plotted against the functional scores in Figure 3.

Correlation coefficients were calculated for each prosthesis comparing the oxford score and the xray score (Where a poor correlation = 0.1-0.3, medium correlation 0.3-0.5, and a good correlation = 0.5-1) [15].

## Results

**Figure 4:**
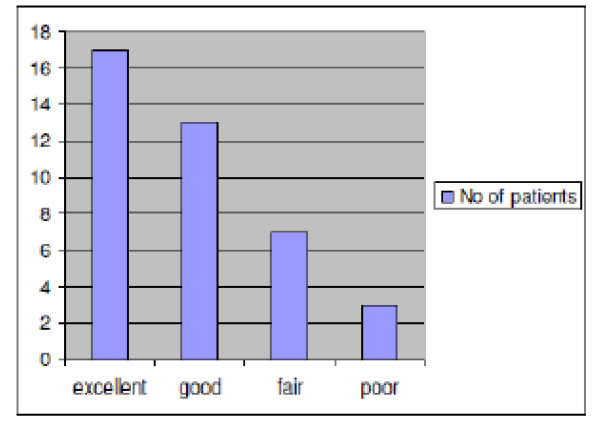
**Distribution of scores in our series**.

**Figure 5:**
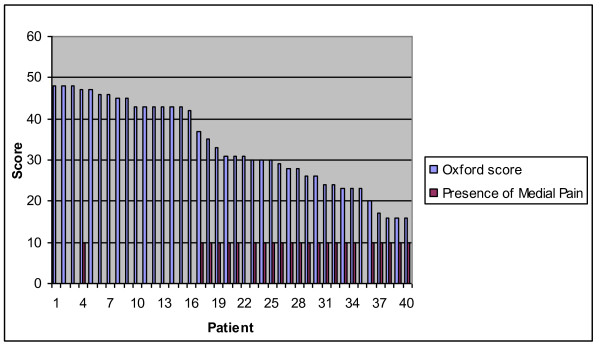
**Plot of Oxford scores against the presence of medial knee pain in each patient**.

38 patients with 40 Oxford knees were available for analysis. Their mean Oxford Functional Score was 32.7, range 16-48, (Figure 4). 17/40 replaced knees (42.5%) scored 'excellent', 13/40 (32.5%) scored 'good', 7/40 (17.5%) 'moderate' and 3/40 (7.5%) were 'poor'. Twenty two of the forty knees exhibited medial knee discomfort or pain (55%) and this symptom was present in 22 of the 24 patients with oxford scores lower than 37 (91.6%) Figure 5.

**Figure 6:**
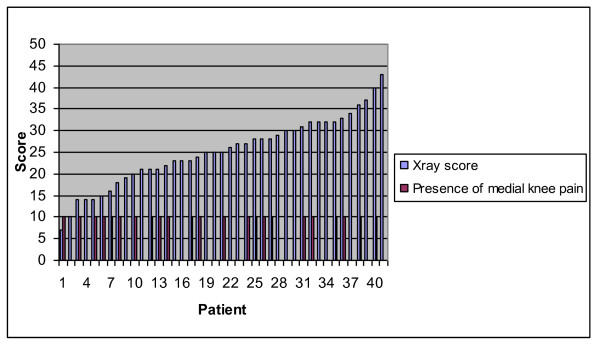
**Plot of Radiographic scores against the presence of medial knee pain for each patient**.

The mean radiographic score was 25.3 (range 7-43), where 0 would signify a perfect radiograph. 6 implants were malpositioned according to the limits for component alignment as suggested in the surgical technique manual. It was noted that the majority of abnormal X-ray criteria arose from apparent varus or valgus placement of the tibial tray or femoral component, and less commonly flexion of the femoral component or posterior tilt of tibial tray. We found no obvious relationship between Xray scores and presence of medial knee pain or discomfort (Figure 6). Excessive medial overhang of the tibial component (more than 2 mm) was seen in 4/40 knees and did not seem to correlate with poor Oxford scores or medial knee discomfort (correlation coefficient = 0.18). In fact the 3 cases with excessive medial overhang of 3 mm, 3 mm and 6 mm had Oxford scores of 45, 43, and 42 respectively.

**Figure 7:**
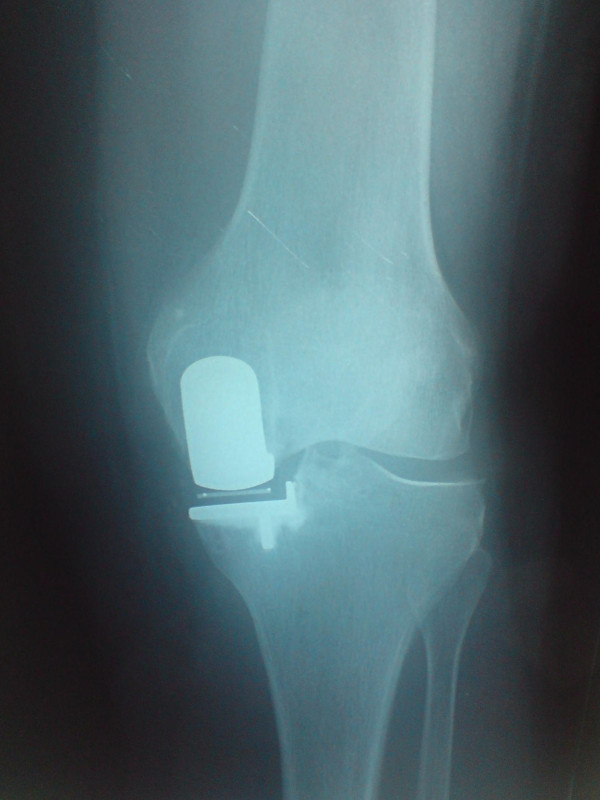
**An example of a knee with a good radiographic score**.

**Figure 8:**
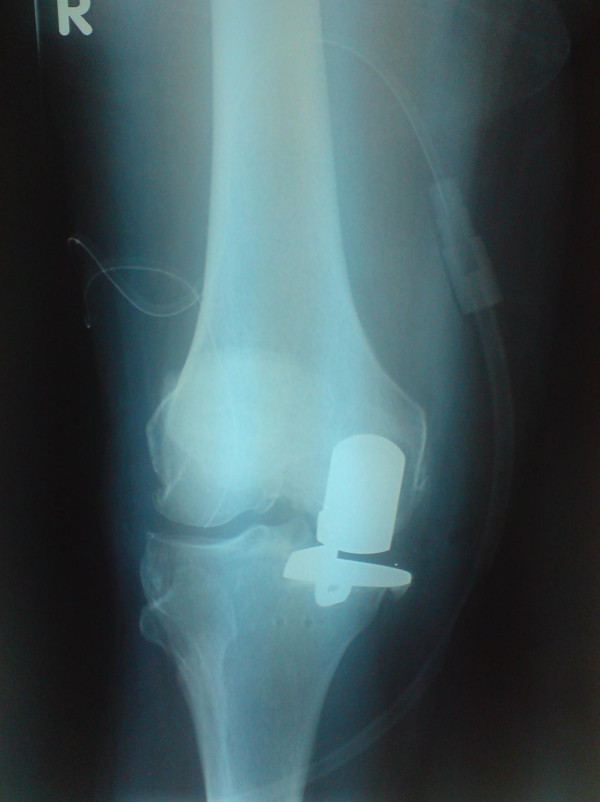
**An example of a knee with a poor radiographic score**.

We found a poor correlation between Oxford Knee Scores and the overall X-ray scores (see Figure 2). For example, patient 1 achieved an Oxford knee score of 48 (best achievable) and scored 30 on X-ray criteria (poor), while another patient achieved 16 on Oxford score (poor), and 16 on X-ray (good alignment). correlation coefficient was 0.107. The closest correlation we found statistically, was a medium correlation, between the varus/valgus positioning of the femoral component and the Oxford score (0.38). Examples of good and poorly positioned prosthesis can be seen in Figures 7 and 8.

We could find no correlation between preoperative arthritis and post operative Oxford scores (correlation coefficient 0.12) or pre op arthritis and Medial knee discomfort (correlation coefficient 0.08).

## Discussion

Several authors have reported good success rates using the Oxford Unicompartmental knee replacement system [14,16]. It has been suggested that results are comparable to that of Total Knee Arthroplasty (TKA) [3].

In our small and retrospective study, 4 of the 48 Oxford unicompartmental knee replacements had been revised within the 4.5 year follow up period and our outcomes in the surviving knees were disappointing compared with other studies [3,4,7,14,16-19], with 7.5% of our patients achieving 'poor' results according to the 'Oxford Knee Scoring' system. Having said this although we were disappointed with our average Oxford core of 33, the average Oxford score following Total knee replacement has been quoted as 34.82 at two years in a recent large study [20].

Our results are similar to those reported by Van Isaker et al, who demonstrated functional results to be poor in 10% of their followed up knees [8], and Cottenie et al [9] in which 6% had poor and 4% fair functional ratings. Both of these studies used the 'Hospital for Special Surgery' score, not the Oxford functional rating system that we used.

In our study four UKAs required revision: two were revised for pain secondary to progressive lateral tibiofemoral compartment degenerative change, one was revised after avascular necrosis developed within the lateral femoral condyle, and one was revised because of persitent and unexplained medial pain, in all cases symptoms resolved with conversion to TKA.

We found little correlation between component mal-positioning and poor oxford scores. This is in keeping with very recent work by the Oxford group who concluded that because of the spherical femoral component, the Oxford UKR is tolerant to femoral mal-alignment of 10° and tibial mal-alignment of 5° [21].

We feel medial knee pain is problematic in this prosthesis. There are several possible aetiologies for medial discomfort including: impingement; medial overhang of the tibial component; cementing errors; aseptic loosening of femur or tibia; soft tissue irritation (MCL, Pes Anserinus); and neuroma formation. Unfortunately there are a group of patients that get unexplained medial pain which is not attributable to any of these factors. Of those with unexplained pain occasionally these will often settle after 1-2y, however it is our experience that an unacceptable number (22/40) persist beyond this time. Our study included only patients of > 3y post op and therefore those 'early settlers' are excluded automatically.

Patients reporting medial knee pain had poorer Oxford scores (Figure 4). 91.6% (22/24) of those with medial pain had scores of 37 or less, as far as we are aware this close correlation has not been previously reported. It is noteworthy that we found a relatively high incidence of medial knee pain despite the fact that phase III Unicompartmental replacements were used.

Although excessive medial overhang of the tibial component (more than 2 mm) was seen in 4/40 knees this did not seem to correlate with poor Oxford scores or medial knee discomfort. This is in keeping with the most recent results reported by Murray et al [22]. They reported that medial overhang of < 3 mm and did not worsen Oxford scores when compared with an overhang of > 3 mm which did have a negative impact on the scores, they did not report an association with medial joint discomfort or pain. It should be noted that in Figure 8 the Radiograph is rotated so the overhang visible is likely to be mostly posteromedial, which *could *be less problematic than direct or anteromedial overhang. This *may *have some bearing on the lack of correlation between overhang and medial pain as some reported overhangs could have been the less significant 'posteromedial' type. This, however, still does not help in our understanding of why medial pain occurs in high numbers of patients (in our study) following Oxford unicompartmental knee replacement.

A large proportion of our patients experienced medial knee pain (more than half). We believe that this medial discomfort does correlate with poorer results, as none of those with scores > 37 complained of the symptom and all those with scores below that did. However it is not the single most important determinant of poor functional results as several patients (18/22 complaining of medial pain) had outcomes which were 'moderate' to 'good'. Is it possible that the presence of medial knee pain is irrelevant to the outcome of these knees? Certainly we do not believe this to be the case as we have found that medial joint discomfort was a common reason for patient dissatisfaction with the Oxford UKA, with one patient requiring revision to TKR (With successful outcome).

There are suggestions that patients with lesser degrees of osteoarthritis preoperatively do not achieve such good results with arthoplasty as those with greater wear. Within our small sample we did not find this to be the case, and furthermore, we did not note a correlation between severity of preoperative osteoarthritis and presence of post op persistent medial discomfort.

There are limitations to our study including being a retrospective review of a small cohort. Due to the fact that we excluded all patients with significant patellofemoral arthritis, we performed very few UKAs (48) when compared with TKAs (around 740) during the period studied and this may, of course, have a significant bearing on our results. It has been suggested that as the Oxford unicompartmental arthroplasty is a demanding procedure that the outcomes are better in units where the operation is being performed frequently [18,23-25]. When the cause for revision of Knee replacement was studied from the New Zealand Joint registry data, it was noted that the early revision rate for the Oxford unicompartmental knee was 2.9 times greater than that for Total knee replacement. However, higher-use surgeons (i.e. those performing one/month or more) had a revision rate comparable to TKA. Those performing > 12 per year had a revision rate of 0.99%, those performing 8-11 per year had revision rates of 4%, those performing 2-7 per year 6.4% and those performing 1 per y had an 8% revision rate [26].

We used standard post operative Xrays to score alignment of prostheses, rather than 'screened' radiographs, and we accept this may affect the calculation of the radiographic scores.

## Conclusion

Our small study demonstrated disappointing medium term results with the 'Oxford Unicompartmental Knee Arthroplasty', 7.5% achieving 'poor' Oxford scores, and around 9% requiring revision within 5 years. We accept that these poor results could be attributable to the relatively low numbers performed in our unit. We also accept that performing unicompartmental replacements more frequently could improve our results, this could be done by extending our indications and ignoring the presence of patellofemoral arthritis (if not clinically symptomatic) as suggested in the new guidelines by the Oxford group.

The vast majority of those patients in our study reporting medial knee pain recorded Oxford scores of < 37, and we feel that the presence of medial knee pain is associated with poorer functional results. Furthermore, it is our experience that this symptom is a common complaint when following up these patients, regardless of the alignment of the prosthesis. Although not formally assessed in this study, we find our patients exhibited significant dissatisfaction with the persistence of medial knee pain post operatively. We also noted no significant correlation between grade of preoperative arthritis and post operative Oxford score or medial knee pain.

Finally, we note that despite current interest in optimising the positioning of UKA to improve functional results, our study failed to demonstrated a correlation between the radiographic alignment of the prosthesis and the patients functional Oxford score.

## Competing interests

The authors declare that they have no competing interests.

## Authors' contributions

All authors were involved with the assessment and subsequent follow up of these patients, and all authors have read and approved the manuscript.
